# (3*Z*,3′*Z*)-3,3′-(3,5-Dimethyl­furan-2,4-diyl)bis­(4-hy­droxy­pent-3-en-2-one)

**DOI:** 10.1107/S1600536812007696

**Published:** 2012-02-24

**Authors:** Mansour S. Al-Said, Mostafa M. Ghorab, Suchada Chantrapromma, Hoong-Kun Fun

**Affiliations:** a Medicinal, Aromatic and Poisonous Plants Research Center (MAPPRC), College of Pharmacy, King Saud University, PO Box 2457, Riyadh 11451, Saudia Arabia; b Crystal Materials Research Unit, Department of Chemistry, Faculty of Science, Prince of Songkla University, Hat-Yai, Songkhla 90112, Thailand; c X-ray Crystallography Unit, School of Physics, Universiti Sains Malaysia, 11800 USM, Penang, Malaysia

## Abstract

In the title mol­ecule, C_16_H_20_O_5_, the two 4-hy­droxy­pent-3-en-2-one units are essentially planar, with r.m.s. deviations of 0.0183 (2) and 0.0134 (2) Å for the non-H atoms, and make dihedral angles of 81.20 (10) and 84.44 (10)° with the central furan ring. The dihedral angle between these two side units is 22.06 (9)°. Two intra­molecular O—H⋯O hydrogen bonds generate two *S*(6) ring motifs. A weak inter­molecular C—H⋯O inter­action is also observed.

## Related literature

For bond-length data, see: Allen *et al.* (1987 ▶). For details of hydrogen-bond motifs, see: Bernstein *et al.* (1995 ▶). For applications of heterocyclic compounds, see: Abdel-Hamid *et al.* (2011 ▶); Alqasoumi *et al.* (2010 ▶); Al-Said *et al.* (2010 ▶, 2011 ▶); Ghorab *et al.* (2001 ▶); Ghorab, Al-Said & El-Hossary (2011 ▶); Ghorab, Ragab *et al.* (2011 ▶, 2012 ▶).
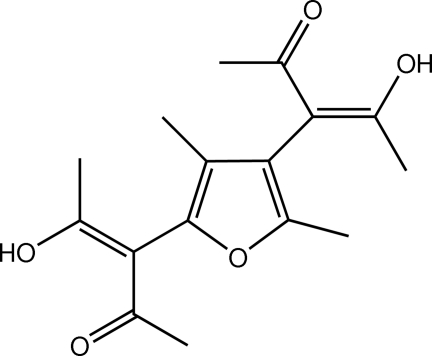


## Experimental

### 

#### Crystal data

C_16_H_20_O_5_
*M* *_r_* = 292.32
Triclinic, 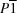

*a* = 7.2645 (2) Å
*b* = 8.5771 (2) Å
*c* = 13.0931 (5) Å
α = 88.384 (2)°
β = 76.390 (2)°
γ = 87.814 (1)°
*V* = 792.17 (4) Å^3^
*Z* = 2
Cu *K*α radiation
μ = 0.75 mm^−1^
*T* = 296 K
0.59 × 0.55 × 0.19 mm

#### Data collection

Bruker SMART APEXII CCD area-detector diffractometer
Absorption correction: multi-scan (*SADABS*; Bruker, 2009 ▶) *T*_min_ = 0.665, *T*_max_ = 0.868
6643 measured reflections
2597 independent reflections
2344 reflections with *I* > 2σ(*I*)
*R*_int_ = 0.023

#### Refinement

*R*[*F* ^2^ > 2σ(*F* ^2^)] = 0.056
*wR*(*F* ^2^) = 0.170
*S* = 1.04
2597 reflections
196 parameters
H-atom parameters constrained
Δρ_max_ = 0.20 e Å^−3^
Δρ_min_ = −0.25 e Å^−3^

### 

Data collection: *APEX2* (Bruker, 2009 ▶); cell refinement: *SAINT* (Bruker, 2009 ▶); data reduction: *SAINT*; program(s) used to solve structure: *SHELXTL* (Sheldrick, 2008 ▶); program(s) used to refine structure: *SHELXTL*; molecular graphics: *SHELXTL*; software used to prepare material for publication: *SHELXTL* and *PLATON* (Spek, 2009 ▶).

## Supplementary Material

Crystal structure: contains datablock(s) global, I. DOI: 10.1107/S1600536812007696/is5075sup1.cif

Structure factors: contains datablock(s) I. DOI: 10.1107/S1600536812007696/is5075Isup2.hkl

Supplementary material file. DOI: 10.1107/S1600536812007696/is5075Isup3.cml

Additional supplementary materials:  crystallographic information; 3D view; checkCIF report

## Figures and Tables

**Table 1:** Hydrogen-bond geometry (Å, °)

*D*—H⋯*A*	*D*—H	H⋯*A*	*D*⋯*A*	*D*—H⋯*A*
O3—H3*A*⋯O2	0.82	1.71	2.457 (3)	150
O5—H5*A*⋯O4	0.82	1.73	2.470 (3)	148
C15—H15*A*⋯O3^i^	0.96	2.60	3.507 (3)	158

Symmetry code: (i) 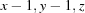.

**Fig. 1.**
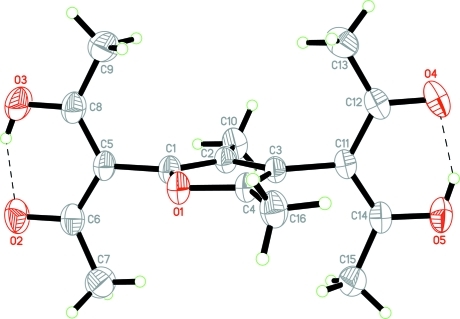
The molecular structure of the title compound, showing 30% probability displacement ellipsoids and the atom-numbering scheme. The O—H···O hydrogen bonds are drawn as dash lines.

**Fig. 2.**
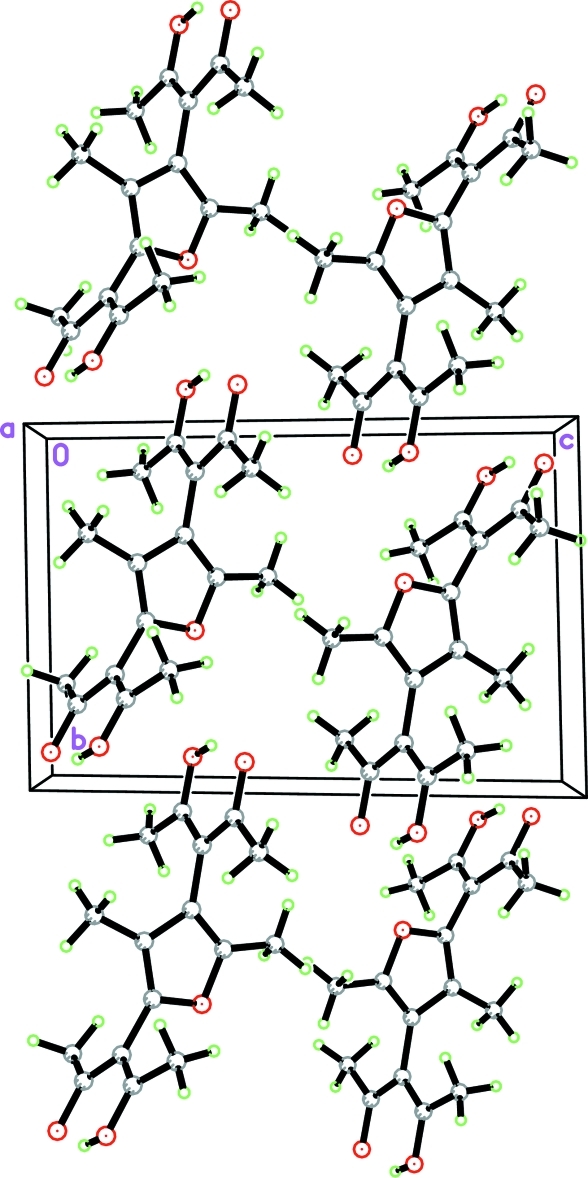
The crystal packing diagram of the title compound, viewed along the a axis.

## supplementary crystallographic information

### Comment

Cancer is a disease of striking significance in the world today. It represents the second leading cause of human mortality after cardiovascular diseases. In order to develop more effective and reliable anticancer agents, a large number of compounds carrying oxygen or nitrogen heterocyclic skeletons have been discovered particularly and many of them exhibited excellent anticancer activities (Alqasoumi *et al.*, 2010; Al-Said *et al.*, 2010; Ghorab *et al.*, 2001; Ghorab, Ragab *et al.*, 2012). On the other hand, furan derivatives are important biologically active compounds showing anticancer activity. From the literature survey, it was found that furan derivatives have been intensively studied for their interesting pharmacological properties such as anticancer activity (Abdel-Hamid *et al.*, 2011). In the light of these facts, and as a continuation of our research (Al-Said *et al.*, 2011; Ghorab, Al-Said & El-Hossary, 2011; Ghorab, Ragab *et al.*, 2011), the present investigation reports the design and synthesis of the title novel furan derivative (I) with the hope that this new compound might show significant anticancer activity. Herein its crystal structure is reported.

In Fig. 1, the molecule of (I), C_16_H_20_O_5_, has a ladder-like structure with the 3,5-dimethylfuran moiety in the middle between the two nearly parallel side chains of 4-hydroxypent-3-en-2-one moieties. The two units of 4-hydroxypent-3-en-2-one are planar with r.m.s. deviations of 0.0183 (2) and 0.0134 (2) Å for the seven non H atoms C5–C9/O2/O3 and C11–C15/O4/O5, respectively. Intramolecular O3—H3···O2 and O5—H5···O4 hydrogen bonds (Table 1) generate two S(6) ring motifs (Bernstein *et al.*, 1995) which help to stabilize the planarity of these units. The C5—C8 [1.403 Å] and C11—C14 [1.386 Å] bond lengths are slightly longer than the usual C═C double bond. However, the angles around atoms C5, C8, C11 and C14 [114.2-123.2 °] indicate the sp^2^ hybridization of these atoms. The furan ring makes the dihedral angles of 81.20 (10) and 84.44 (10)° with the mean planes of C5–C9/O2/O3 and C11–C15/O4/O5, respectively. Whereas the dihedral angle between these two planes is 22.06 (9)°. The two methyl groups are co-planar with the furan ring with an r.m.s. deviation of 0.0143 (2) Å. The bond distances in (I) are within normal ranges (Allen *et al.*, 1987). The crystal is consolidated by weak C···H···O interactions (Table 1). Even there is no hydrogen bond in the crystal packing but the crystal packing was shown in Fig. 2 to show the arrangement of the molecules.

### Experimental

Ethanol (30 ml) was converted to sodium ethoxide by portionwise addition of sodium (0.46 g, 0.02 mole) before a solution of diethyl oxalate (2.92 g, 0.02 mole) and 3-acetyl-2,5-dimethylfuran (1.38 g, 0.01 mole) in ethanol (30 ml) was added dropwise at room temperature. The reaction mixture was heated under reflux for 4 h. After cooling, the solvent was removed and the residue was taken up in water (100 ml) and acidified with concentrated HCl (3 ml). The aqueous mixture was extracted with diethylether (3 × 100 ml), dried over MgSO_4_. The obtained solid was recrystallized from ethanol to give the title compound. Colorless block-shaped single crystals suitable for an *X*-ray structural analysis was obtained by slowly evaporating from ethanol at room temperature.

### Refinement

All H atoms were placed in calculated positions with d(O—H) = 0.82 Å and d(C—H) = 0.96 Å. The *U*_iso_(H) values were constrained to be 1.2*U*_eq_ of the carrier atom for hydroxy H atoms and 1.5*U*_eq_ for the methyl H atoms. A rotating group model was used for the methyl groups.

### Crystal data

**Table:**

C_16_H_20_O_5_	*Z* = 2
*M**_r_* = 292.32	*F*(000) = 312
Triclinic, *P*1	*D*_x_ = 1.226 Mg m^−^^3^
Hall symbol: -P 1	Cu *K*α radiation, λ = 1.54178 Å
*a* = 7.2645 (2) Å	Cell parameters from 2597 reflections
*b* = 8.5771 (2) Å	θ = 5.2–65.0°
*c* = 13.0931 (5) Å	µ = 0.75 mm^−^^1^
α = 88.384 (2)°	*T* = 296 K
β = 76.390 (2)°	Block, colorless
γ = 87.814 (1)°	0.59 × 0.55 × 0.19 mm
*V* = 792.17 (4) Å^3^	

### Data collection

**Table:**

Bruker SMART APEXII CCD area-detector diffractometer	2597 independent reflections
Radiation source: fine-focus sealed tube	2344 reflections with *I* > 2σ(*I*)
Graphite monochromator	*R*_int_ = 0.023
φ and ω scans	θ_max_ = 65.0°, θ_min_ = 5.2°
Absorption correction: multi-scan (*SADABS*; Bruker, 2009)	*h* = −8→7
*T*_min_ = 0.665, *T*_max_ = 0.868	*k* = −10→10
6643 measured reflections	*l* = −15→15

### Refinement

**Table:**

Refinement on *F*^2^	Primary atom site location: structure-invariant direct methods
Least-squares matrix: full	Secondary atom site location: difference Fourier map
*R*[*F*^2^ > 2σ(*F*^2^)] = 0.056	Hydrogen site location: inferred from neighbouring sites
*wR*(*F*^2^) = 0.170	H-atom parameters constrained
*S* = 1.04	*w* = 1/[σ^2^(*F*_o_^2^) + (0.104*P*)^2^ + 0.160*P*] where *P* = (*F*_o_^2^ + 2*F*_c_^2^)/3
2597 reflections	(Δ/σ)_max_ = 0.001
196 parameters	Δρ_max_ = 0.20 e Å^−^^3^
0 restraints	Δρ_min_ = −0.25 e Å^−^^3^

### Special details

**Table:**

Geometry. All esds (except the esd in the dihedral angle between two l.s. planes) are estimated using the full covariance matrix. The cell esds are taken into account individually in the estimation of esds in distances, angles and torsion angles; correlations between esds in cell parameters are only used when they are defined by crystal symmetry. An approximate (isotropic) treatment of cell esds is used for estimating esds involving l.s. planes.
Refinement. Refinement of F^2^ against ALL reflections. The weighted R-factor wR and goodness of fit S are based on F^2^, conventional R-factors R are based on F, with F set to zero for negative F^2^. The threshold expression of F^2^ > 2sigma(F^2^) is used only for calculating R-factors(gt) etc. and is not relevant to the choice of reflections for refinement. R-factors based on F^2^ are statistically about twice as large as those based on F, and R- factors based on ALL data will be even larger.

### Fractional atomic coordinates and isotropic or equivalent isotropic displacement parameters (Å^2^)

**Table:**

	*x*	*y*	*z*	*U*_iso_*/*U*_eq_	
O1	0.02411 (17)	0.56371 (13)	0.31031 (9)	0.0579 (4)	
O2	0.2498 (3)	0.88634 (18)	0.05070 (13)	0.0854 (5)	
O3	0.5019 (2)	0.8614 (2)	0.14714 (14)	0.0912 (5)	
H3A	0.4399	0.8966	0.1064	0.137*	
O4	0.1552 (3)	−0.08978 (17)	0.39756 (13)	0.0854 (5)	
O5	−0.0968 (2)	−0.09275 (16)	0.29980 (13)	0.0825 (5)	
H5A	−0.0240	−0.1262	0.3351	0.124*	
C1	0.1759 (3)	0.5388 (2)	0.22504 (14)	0.0547 (4)	
C2	0.2125 (2)	0.38426 (19)	0.21246 (13)	0.0538 (4)	
C3	0.0787 (2)	0.30746 (19)	0.29583 (13)	0.0508 (4)	
C4	−0.0310 (3)	0.4202 (2)	0.35119 (14)	0.0538 (4)	
C5	0.2626 (3)	0.6793 (2)	0.16975 (14)	0.0583 (5)	
C6	0.1785 (3)	0.7605 (2)	0.09731 (15)	0.0663 (5)	
C7	0.0029 (4)	0.7092 (3)	0.0709 (2)	0.0870 (7)	
H7A	−0.0877	0.7950	0.0782	0.131*	
H7B	−0.0491	0.6254	0.1177	0.131*	
H7C	0.0318	0.6739	−0.0002	0.131*	
C8	0.4249 (3)	0.7383 (2)	0.19331 (17)	0.0690 (5)	
C9	0.5166 (3)	0.6632 (3)	0.2732 (2)	0.0892 (7)	
H9A	0.6096	0.7309	0.2872	0.134*	
H9B	0.5770	0.5661	0.2470	0.134*	
H9C	0.4224	0.6442	0.3368	0.134*	
C10	0.3607 (3)	0.3067 (3)	0.12838 (17)	0.0742 (6)	
H10A	0.4233	0.3843	0.0795	0.111*	
H10B	0.3021	0.2346	0.0921	0.111*	
H10C	0.4518	0.2516	0.1596	0.111*	
C11	0.0602 (3)	0.13679 (19)	0.31678 (13)	0.0547 (4)	
C12	0.1725 (3)	0.0557 (2)	0.37746 (15)	0.0659 (5)	
C13	0.3133 (4)	0.1371 (3)	0.4210 (2)	0.0940 (8)	
H13A	0.3939	0.0613	0.4460	0.141*	
H13B	0.2482	0.2012	0.4780	0.141*	
H13C	0.3887	0.2012	0.3669	0.141*	
C14	−0.0705 (3)	0.0548 (2)	0.27961 (15)	0.0632 (5)	
C15	−0.1914 (4)	0.1271 (3)	0.2129 (2)	0.0890 (7)	
H15A	−0.2962	0.0616	0.2140	0.134*	
H15B	−0.1179	0.1386	0.1420	0.134*	
H15C	−0.2383	0.2277	0.2395	0.134*	
C16	−0.1966 (3)	0.4172 (2)	0.44325 (16)	0.0697 (6)	
H16A	−0.2166	0.3117	0.4688	0.105*	
H16B	−0.3075	0.4577	0.4224	0.105*	
H16C	−0.1724	0.4801	0.4979	0.105*	

### Atomic displacement parameters (Å^2^)

**Table:**

	*U*^11^	*U*^22^	*U*^33^	*U*^12^	*U*^13^	*U*^23^
O1	0.0659 (8)	0.0415 (6)	0.0592 (7)	−0.0044 (5)	−0.0001 (6)	0.0031 (5)
O2	0.1097 (12)	0.0637 (9)	0.0794 (10)	−0.0191 (8)	−0.0158 (9)	0.0265 (7)
O3	0.0894 (11)	0.0805 (11)	0.1025 (12)	−0.0404 (9)	−0.0176 (9)	0.0250 (9)
O4	0.1135 (13)	0.0554 (9)	0.0822 (10)	0.0142 (8)	−0.0179 (9)	0.0180 (7)
O5	0.1042 (12)	0.0466 (8)	0.0954 (11)	−0.0176 (7)	−0.0198 (9)	0.0084 (7)
C1	0.0564 (10)	0.0486 (9)	0.0544 (9)	−0.0075 (7)	−0.0030 (7)	0.0045 (7)
C2	0.0551 (9)	0.0474 (9)	0.0555 (9)	−0.0046 (7)	−0.0065 (7)	0.0054 (7)
C3	0.0552 (9)	0.0426 (9)	0.0536 (9)	−0.0053 (7)	−0.0105 (7)	0.0059 (7)
C4	0.0601 (10)	0.0446 (9)	0.0537 (9)	−0.0077 (7)	−0.0071 (7)	0.0064 (7)
C5	0.0633 (11)	0.0469 (9)	0.0587 (10)	−0.0095 (8)	−0.0013 (8)	0.0044 (7)
C6	0.0791 (13)	0.0561 (11)	0.0582 (11)	−0.0090 (9)	−0.0047 (9)	0.0049 (8)
C7	0.0965 (17)	0.0908 (17)	0.0779 (14)	−0.0173 (13)	−0.0279 (12)	0.0123 (12)
C8	0.0658 (12)	0.0605 (11)	0.0736 (12)	−0.0148 (9)	−0.0010 (9)	0.0074 (9)
C9	0.0727 (14)	0.0967 (18)	0.0993 (17)	−0.0199 (13)	−0.0222 (12)	0.0210 (14)
C10	0.0751 (13)	0.0631 (12)	0.0720 (13)	0.0052 (10)	0.0058 (10)	0.0032 (9)
C11	0.0643 (10)	0.0420 (9)	0.0531 (9)	−0.0006 (7)	−0.0054 (7)	0.0060 (7)
C12	0.0771 (12)	0.0568 (11)	0.0590 (11)	0.0070 (9)	−0.0090 (9)	0.0088 (8)
C13	0.0952 (17)	0.0977 (18)	0.0954 (18)	−0.0016 (14)	−0.0378 (14)	0.0198 (14)
C14	0.0750 (12)	0.0458 (10)	0.0640 (11)	−0.0068 (8)	−0.0064 (9)	0.0040 (8)
C15	0.0996 (18)	0.0742 (15)	0.1048 (18)	−0.0193 (13)	−0.0461 (15)	0.0119 (13)
C16	0.0741 (13)	0.0586 (11)	0.0650 (11)	−0.0026 (9)	0.0056 (9)	0.0070 (9)

### Geometric parameters (Å, º)

**Table:**

O1—C4	1.364 (2)	C8—C9	1.486 (3)
O1—C1	1.387 (2)	C9—H9A	0.9600
O2—C6	1.289 (2)	C9—H9B	0.9600
O3—C8	1.281 (2)	C9—H9C	0.9600
O3—H3A	0.8200	C10—H10A	0.9600
O4—C12	1.272 (2)	C10—H10B	0.9600
O5—C14	1.297 (2)	C10—H10C	0.9600
O5—H5A	0.8200	C11—C14	1.386 (3)
C1—C2	1.349 (2)	C11—C12	1.418 (3)
C1—C5	1.472 (2)	C12—C13	1.486 (3)
C2—C3	1.442 (2)	C13—H13A	0.9600
C2—C10	1.497 (2)	C13—H13B	0.9600
C3—C4	1.344 (2)	C13—H13C	0.9600
C3—C11	1.487 (2)	C14—C15	1.486 (3)
C4—C16	1.490 (2)	C15—H15A	0.9600
C5—C6	1.401 (3)	C15—H15B	0.9600
C5—C8	1.403 (3)	C15—H15C	0.9600
C6—C7	1.483 (3)	C16—H16A	0.9600
C7—H7A	0.9600	C16—H16B	0.9600
C7—H7B	0.9600	C16—H16C	0.9600
C7—H7C	0.9600		
			
C4—O1—C1	106.75 (13)	H9B—C9—H9C	109.5
C8—O3—H3A	109.5	C2—C10—H10A	109.5
C14—O5—H5A	109.5	C2—C10—H10B	109.5
C2—C1—O1	109.76 (15)	H10A—C10—H10B	109.5
C2—C1—C5	133.94 (17)	C2—C10—H10C	109.5
O1—C1—C5	116.29 (15)	H10A—C10—H10C	109.5
C1—C2—C3	106.28 (15)	H10B—C10—H10C	109.5
C1—C2—C10	127.22 (17)	C14—C11—C12	118.74 (17)
C3—C2—C10	126.50 (16)	C14—C11—C3	120.62 (16)
C4—C3—C2	106.76 (15)	C12—C11—C3	120.62 (17)
C4—C3—C11	125.75 (16)	O4—C12—C11	121.2 (2)
C2—C3—C11	127.48 (15)	O4—C12—C13	117.37 (19)
C3—C4—O1	110.43 (15)	C11—C12—C13	121.47 (19)
C3—C4—C16	132.98 (16)	C12—C13—H13A	109.5
O1—C4—C16	116.57 (15)	C12—C13—H13B	109.5
C6—C5—C8	119.11 (17)	H13A—C13—H13B	109.5
C6—C5—C1	120.53 (17)	C12—C13—H13C	109.5
C8—C5—C1	120.27 (17)	H13A—C13—H13C	109.5
O2—C6—C5	121.16 (19)	H13B—C13—H13C	109.5
O2—C6—C7	116.34 (19)	O5—C14—C11	122.60 (19)
C5—C6—C7	122.50 (18)	O5—C14—C15	114.18 (19)
C6—C7—H7A	109.5	C11—C14—C15	123.22 (17)
C6—C7—H7B	109.5	C14—C15—H15A	109.5
H7A—C7—H7B	109.5	C14—C15—H15B	109.5
C6—C7—H7C	109.5	H15A—C15—H15B	109.5
H7A—C7—H7C	109.5	C14—C15—H15C	109.5
H7B—C7—H7C	109.5	H15A—C15—H15C	109.5
O3—C8—C5	121.5 (2)	H15B—C15—H15C	109.5
O3—C8—C9	116.2 (2)	C4—C16—H16A	109.5
C5—C8—C9	122.28 (18)	C4—C16—H16B	109.5
C8—C9—H9A	109.5	H16A—C16—H16B	109.5
C8—C9—H9B	109.5	C4—C16—H16C	109.5
H9A—C9—H9B	109.5	H16A—C16—H16C	109.5
C8—C9—H9C	109.5	H16B—C16—H16C	109.5
H9A—C9—H9C	109.5		
			
C4—O1—C1—C2	0.7 (2)	C8—C5—C6—O2	1.4 (3)
C4—O1—C1—C5	−178.48 (15)	C1—C5—C6—O2	177.88 (17)
O1—C1—C2—C3	−1.2 (2)	C8—C5—C6—C7	−177.8 (2)
C5—C1—C2—C3	177.8 (2)	C1—C5—C6—C7	−1.2 (3)
O1—C1—C2—C10	178.20 (18)	C6—C5—C8—O3	−2.7 (3)
C5—C1—C2—C10	−2.8 (4)	C1—C5—C8—O3	−179.23 (18)
C1—C2—C3—C4	1.2 (2)	C6—C5—C8—C9	176.9 (2)
C10—C2—C3—C4	−178.19 (19)	C1—C5—C8—C9	0.4 (3)
C1—C2—C3—C11	−179.96 (17)	C4—C3—C11—C14	82.7 (2)
C10—C2—C3—C11	0.6 (3)	C2—C3—C11—C14	−95.9 (2)
C2—C3—C4—O1	−0.8 (2)	C4—C3—C11—C12	−95.7 (2)
C11—C3—C4—O1	−179.63 (15)	C2—C3—C11—C12	85.7 (2)
C2—C3—C4—C16	177.7 (2)	C14—C11—C12—O4	0.2 (3)
C11—C3—C4—C16	−1.1 (3)	C3—C11—C12—O4	178.64 (17)
C1—O1—C4—C3	0.1 (2)	C14—C11—C12—C13	−179.13 (19)
C1—O1—C4—C16	−178.72 (17)	C3—C11—C12—C13	−0.7 (3)
C2—C1—C5—C6	101.3 (3)	C12—C11—C14—O5	1.4 (3)
O1—C1—C5—C6	−79.7 (2)	C3—C11—C14—O5	−177.04 (17)
C2—C1—C5—C8	−82.2 (3)	C12—C11—C14—C15	−178.23 (19)
O1—C1—C5—C8	96.8 (2)	C3—C11—C14—C15	3.4 (3)

### Hydrogen-bond geometry (Å, º)

**Table:**

*D*—H···*A*	*D*—H	H···*A*	*D*···*A*	*D*—H···*A*
O3—H3*A*···O2	0.82	1.71	2.457 (3)	150
O5—H5*A*···O4	0.82	1.73	2.470 (3)	148
C15—H15*A*···O3^i^	0.96	2.60	3.507 (3)	158

Symmetry code: (i) *x*−1, *y*−1, *z*.
